# Draft genome of a commonly misdiagnosed multidrug resistant pathogen *Candida auris*

**DOI:** 10.1186/s12864-015-1863-z

**Published:** 2015-09-07

**Authors:** Sharanya Chatterjee, Shuba Varshini Alampalli, Rishi Kumar Nageshan, Sivarajan T. Chettiar, Sangeeta Joshi, Utpal S. Tatu

**Affiliations:** Department of Biochemistry, Indian Institute of Science, Bengaluru, Karnataka India 560012; Manipal Hospital, Bengaluru, Karnataka India

**Keywords:** Nosocomial infections, Drug resistance, Virulence, Fungemia, Misdiagnosis, *Candida haemulonii*, Next Generation Sequencing (NGS)

## Abstract

**Background:**

*Candida auris* is a multidrug resistant, emerging agent of fungemia in humans. Its actual global distribution remains obscure as the current commercial methods of clinical diagnosis misidentify it as *C. haemulonii*. Here we report the first draft genome of *C. auris* to explore the genomic basis of virulence and unique differences that could be employed for differential diagnosis.

**Results:**

More than 99.5 % of the *C. auris* genomic reads did not align to the current whole (or draft) genome sequences of *Candida albicans*, *Candida lusitaniae*, *Candida glabrata* and *Saccharomyces cerevisiae*; thereby indicating its divergence from the active *Candida* clade. The genome spans around 12.49 Mb with 8527 predicted genes. Functional annotation revealed that among the sequenced *Candida* species, it is closest to the hemiascomycete species *Clavispora lusitaniae*. Comparison with the well-studied species *Candida albicans* showed that it shares significant virulence attributes with other pathogenic *Candida* species such as oligopeptide transporters, mannosyl transfersases, secreted proteases and genes involved in biofilm formation. We also identified a plethora of transporters belonging to the ABC and major facilitator superfamily along with known MDR transcription factors which explained its high tolerance to antifungal drugs.

**Conclusions:**

Our study emphasizes an urgent need for accurate fungal screening methods such as PCR and electrophoretic karyotyping to ensure proper management of fungemia. Our work highlights the potential genetic mechanisms involved in virulence and pathogenicity of an important emerging human pathogen namely *C. auris*. Owing to its diversity at the genomic scale; we expect the genome sequence to be a useful resource to map species specific differences that will help develop accurate diagnostic markers and better drug targets.

**Electronic supplementary material:**

The online version of this article (doi:10.1186/s12864-015-1863-z) contains supplementary material, which is available to authorized users.

## Background

Hospital acquired infection (HAI) also known as nosocomial infections are gaining momentum and Centre for Disease control USA, estimates about 99,000 deaths a year due to infections acquired from hospital [1]. HAI are caused by organisms that include bacteria and fungi entering via surgical sites, urinary and other catheters. Nosocomial associated invasive fungal diseases are of public health concern and candidemia is becoming very prevalent in European countries [2]. The fourth most common cause of bloodstream infection is *Candida*, accounting for more than 85 % of all fungemias in USA and Europe [3, 4]. However the rising number of immunocompromised people, unwarranted use of multiple broad spectrum antibiotics and the advent of implanted medical devices [5] has paved the way for rare non albicans *Candida* species [6] as agents of invasive mycoses and nosocomial bloodstream infections [7]. Recently invasive non *albicans* candidiasis cases have been reported from many parts of the world [8–11]. Among the non *albicans Candida* species, *C. tropicalis* and *C. glabrata* have emerged as important opportunistic pathogens [12].

Most recently, species belonging to the *Candida haemulonii* complex [13] has been described as important agent of fungemia with a significant global distribution [14, 15] and Lehman *et al.* [16] categorised these species belonging to *C. haemulonii* into two genetically distinct groups. Furthermore infections caused by two phenotypically related species – *C. pseudohaemulonii* [17] and *C. auris* are on the rise [18]. First described in 2009 by Satoh *et al.* [19] in a Japanese patient, it is striking to see the aggressive pace at which *C. auris* has expanded its clinical spectrum worldwide from minor cases of superficial infections such as ear canal infections to highly invasive cases of bloodstream infections [20]. Previous studies [21] as well as our study demonstrate that all these clinical isolates have a precociously high tolerance to AmphotericinB (AmB) [22] and Fluconozole (Fcz) [14, 15], the first line treatment antifungals. Even more concerning is the rapid emergence of resistance to echinocandins [23], the newest class of antifungals which may leave no treatment option available leading to clinical failure.

Many pathogenic species within the *Candida* clade such as *Candida albicans* and *Candida glabrata* have been extensively studied at the genome level, while emerging fungal pathogens *Candida auris* and *Candida haemulonii* remains unexplored. The basic characteristics of the genome of *C. auris* was recently made available [24]. However detailed information regarding the genome architecture, virulence and mechanisms of multidrug resistance of these emerging novel complexes of pathogenic yeasts are lacking. Furthermore, the commercial automated systems routinely fail to identify *C. auris* correctly; thereby its actual occurrence is underreported. Even more alarming is the fact that misdiagnosis may lead to incorrect treatment or delay of proper treatment, thereby increasing the chances of fatalities. As expected, *C. auris* fungemia is associated with a high mortality rate (66 %) and therapeutic failure [25]. It also does not exhibit the known attributes responsible for virulence in *Candida* species such as hyphae formation and the cells are much smaller in size than that of *C. albicans* (Additional file 1: Figure S2). Towards understanding the basic biology of the multidrug resistant pathogen, we have carried out whole genome sequencing of a multidrug resistant clinical isolate of *C. auris* using Illumina sequencing technology and report that *C. auris* has a highly divergent genome. Analysis using *C. albicans* as a reference genome revealed a set of orthologs such as drug transporters, oligopeptide transporters, secreted proteinases and mannosyl transferases which may play a role in virulence and drug resistance. However most of the genome is uncharacterized and we speculate that some of these hypothetical proteins may be involved in species specific characteristics which promote its aggressiveness as a pathogen.

## Results and discussion

### Clinical isolates of *Candida* show multi drug resistance

**Fig. 1:**
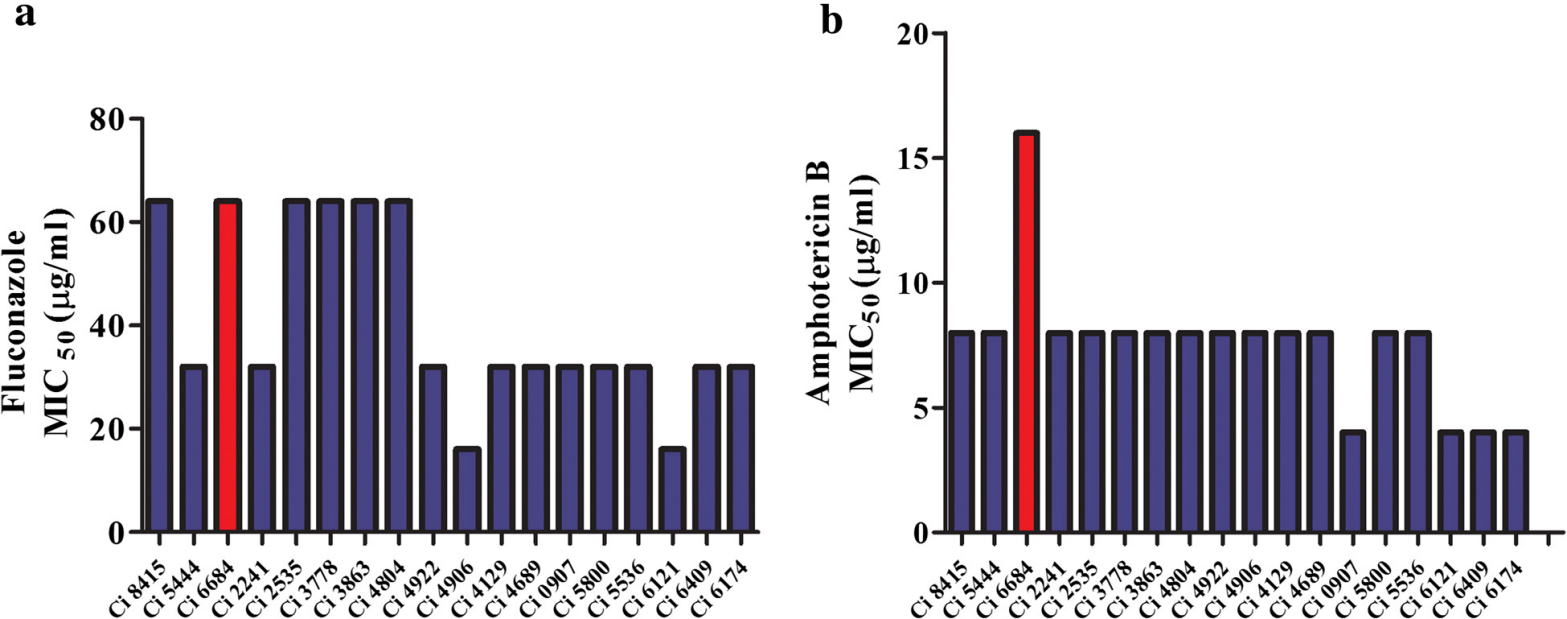
*In vitro* antifungal susceptibility testing of clinical isolates of *Candida:* All the isolates were identified as *C. haemulonii* by Vitek2. Susceptibility testing was done by broth microdilution method at 37 °C for 48 h as mentioned in materials and methods. **a** Comparison of MIC_50_ values of all isolates for Fcz indicates all clinical isolates have MIC range of 32–64 μg/ml. **b** Comparison of MIC_50_ values of all isolates for AmB shows that all clinical isolates are resistant to AmB. *Candida* isolate Ci 6684 has the highest MIC_50_ value of > = 16 μg/ml

With the background of the growing incidences of candidiasis we have determined the hierarchy of the causative *Candida* species from clinical cases of invasive non-albicans candidiasis. In collaboration with Manipal Hospital, Bengaluru we have screened clinical samples from invasive cases of Candidiasis (Additional file 2: Table S1). Identification of the isolates was done by Vitek2 (bioMerieux, Marcy, I’Etoile, France) performed at Manipal Hospital. We saw a significant increase in the frequency of Candidiasis from 2012 to 2014 and we also found that non albicans *Candida* species are occupying the centre stage in such infections. Case reports from bloodstream infections revealed that in 2012, 24.3 % of infections were caused by *C. albicans* and *C. tropicalis*. However in 2014, 38.3 % of the cases were reported to be caused by *C. haemulonii*. Both *C. albicans* and *C. tropicalis* were susceptible to the commonly used antifungals AmB and Fcz (Table 1). However, all the clinical isolates identified as *C. haemulonii* showed increased tolerance to both Fcz and AmB. These isolates are referred as *Candida* isolates (Ci) henceforth. As shown in Fig. 1, the isolates had MIC_50_ value of >32 μg/mL and >7 μg/mL for Fcz and AmB (Fig. 1a,b) respectively. Since the patients were never administered AmB previously, it is difficult to comment that AmB resistance in these set of clinical isolates was inherent or acquired. However all these isolates were susceptible to caspofungin, the newer class of antifungal drugs- echinocandins (data not shown). The isolate Ci 6684 which showed resistance to both AmB and FcZ with highest MIC_50_ values was used for further analysis. The antifungal susceptibility profile of Ci 6684 is presented in Table 2.

**Table 1:** *In vitro* antifungal susceptibility pattern of pathogenic *Candida* clinical isolates (from bloodstream) to the most commonly used drugs belonging to the four different classes of antifungals

Antifungal drugs	Species (n)					
	MIC_50_ range (μg/ml)					
	Clinical isolates_ Ch^a^ (34)	*C. albicans* (20)	*C. tropicalis* (34)	*C. glabrata* (9)	*C. lusitaniae* (2)	*C. parapsilosis* (23)
Fluconazole	16–64	1	1	0.5–1	0.5–1	≤1
Amphotericin B	4–16	0.25	0.25	0.25–0.5	0.12–0.25	0.25–0.5
Flucytosine	0.25	≤1	≤1	≤1	≤1	≤1
Caspofungin	0.25	0.25	0.25	0.12	0.12	0.25–1

^a^All these isolates were identified as *C. haemulonii* by Vitek2, which routinely fails to identify closely related species such as *C. auris*

### Complete genome sequence of the clinical isolate Ci 6684

We sequenced the genome of Ci 6684 using Illumina sequencing technology. A high-quality reference genome using Illumina reads was assembled *de novo* as described in Methods (Additional file 3: Figure S1). The assembled draft genome of Ci 6684 comprises 99 scaffolds with an estimated genome size of 12,498,766 bp, 44.53 % GC and 1.327 % Ns. The average base is found in the scaffold with a scaffold N_50_ of 279 Kb. A total of 8358 protein coding genes, 7 rRNAs and 189 tRNAs were predicting using different tools (Description in Methods and Additional file 3: Figure S1).

The basic annotation of 8358 predicted protein coding genes were done using blastp against current RefSeq fungal protein database and protein NR database. 5175 proteins found orthologs with a mean query coverage of 94.68 % (40–100 %), mean identity of 60.73 % (21.72–100 %) and E-value > e^−10^. 42.38 % of Ci 6684 proteins were orthologous to *C. lusitaniae* ATCC 42720*.* However majority of the proteins were assigned as hypothetical, since the closely related *Candida* species *C. lusitaniae* protein database have also annotated those similar proteins to be hypothetical/ functionally uncharacterized. Ci 6684 was found to be diploid with a similar FACS profile to that of *C. albicans* SC-5314 by flow cytometric analysis as shown in Additional file 4: Figure S3. Table 3 summarizes the general features of the genome of Ci 6684 along with known pathogenic *Candida* species. The average size (bp) of coding sequence domain (CDS) of Ci 6684 seems to be least, whilst the intergenic distance (bp) is similar to that of other species.

**Table 2:** *In vitro* antifungal susceptibility profile of *Candida* clinical isolate Ci 6684

Drug	MIC_50_ (μg/ml)	Susceptibility
Fluconazole	64	R
Amphotericin B	16	R
Flucytosine	1	S
Caspofungin	0.25	S

**Table 3:** General features of *Candida* species and clinical isolate Ci 6684 genome

Organism	Size (Mb)	Number of chromosomes (or scaffolds)	GC content (%)	Number of genes	Average CDS size (bp)	Average intergenic distance (bp)
*C. albicans* SC5314	28.6*	17	33.43	12869	1456.39	859.23
*C. dubliniensis* CD36	14.6	8	33.25	5992	1522.16	8357.8
*C. orthopsilosis* Co 90–125	12.66	8	36.93	5766	1491.86	4945.93
*C. tropicalis* MYA-3404	14.6	23	33.01	6258	1453.47	894.8
*C. guilliermondii* ATCC 6260	10.61	9	43.62	5920	1401.41	427.87
*C. lusitaniae* ATCC 42720	12.1	9	44.37	5941	1387.45	774.18
*C. glabrata* CBS 138	12.3	14	38.62	5235	1526.93	773.63
*S. cerevisiae* S288C	12.2	17	38.15	5916	1485.5	435.79
Ci 6684	12.5	99	44.53	8358	1024.55	828.8395

*Candida albicans SC-5314 used is assembly number 22 and is shown with a diploid set of chromosomes and genes. Rest of the *Candida* species are from the current genomic data available at CGD and Broad Institute. ND - not determined. All decimals are rounded off at the second digit

### Phylogenetic analysis reveals Ci 6684 is closely related to *Candida auris*

**Fig. 2:**
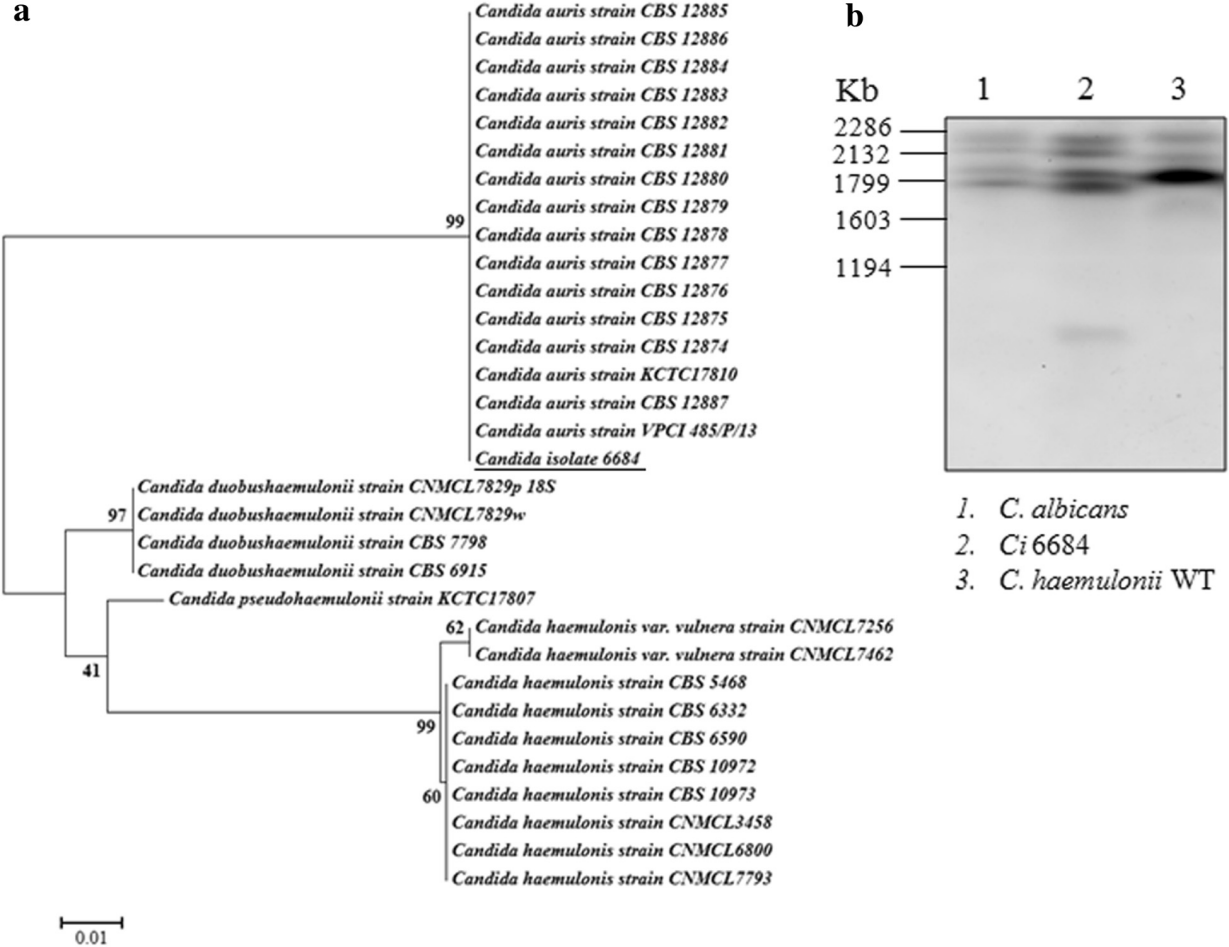
ITS phylogeny and electrophoretic karyotyping reveals that Ci 6684 belongs to *C. auris clade*. **a** Phylogenetic tree based on the partial sequence of 18 s rRNA, ITS1, 5.8 s rRNA complete sequence, ITS2 and 28 s rRNA partial sequences of species belonging to the *C. haemulonii* complex and related *Candida* species. Our isolate Ci 6684 is related to *C. auris* as it falls in the same clade. **b** Electrophoretic karyotyping shows 5 bands similar to those reported for *C. auris* by Oh J B *et al.*, where they reported genotypic relatedness between *C. haemulonii* and closely related species. *C. albicans* (*lane 1*) shows four distinct bands. Corresponding *C. auris* lane (*lane 2*) shows five bands

Phylogenetic tree based on the partial sequence of 18 s rRNA, ITS1, 5.8 s rRNA complete sequence, ITS2 and 28 s rRNA partial sequence revealed that Ci 6684 belongs to *Candida auris* clade of Korean and Indian isolates with 99 % bootstrapped confidence (Fig. 2a). To further confirm its origin, we performed multiple sequence alignment with the Indian *C.* auris isolates and found complete conservation of rRNA and ITS sequences (Additional file 5). The same isolate was also able to grow at 40 °C and 42 °C as reported for *C. auris* but not for *C. haemulonii* [20]*.* Electrophoretic karyotyping by PFGE of Ci6684 yielded 5 bands and the pattern was similar to that reported previously for *C. auris* [26] (Fig. 2b). Because diagnostic laboratories rely only on automated systems like Vitek 2 or APIC20C which routinely identifies *C. auris* as *C. haemulonii* or *C. famata*, the actual occurrence of *C. auris* fungemia is under reported [25, 27, 28]. Our results emphasize the need to develop accurate species identification system based on molecular typing methods to ensure proper management of fungemia. Another recently developed method for identifying *C. auris* by MALDI-TOF has also been reported [29]. Henceforth Ci 6684 will be referred to *C. auris* 6684 in the remaining study.

**Fig. 3:**
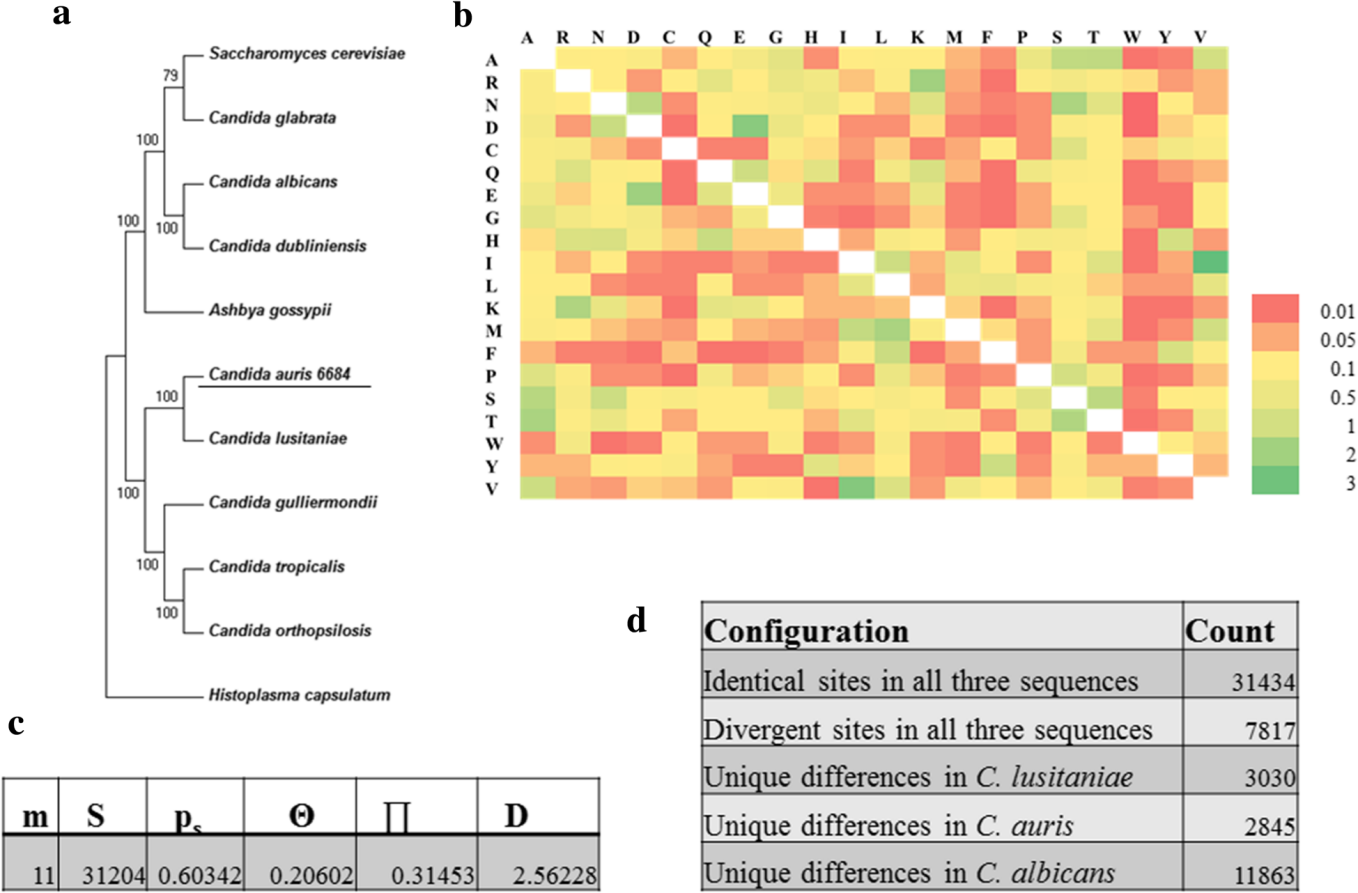
Evolutionary position of *Candida auris* isolate 6684 in the pathogenic fungal tree. **a** Phylogenetic tree based on orthologs of 95 conserved proteins from 11 pathogenic species under the phylum *Ascomycota.* **b** Amino acid substitution matrix by maximum likelihood estimation. **c** Tajima’s neutrality test indicates low level of polymorphism in the house keeping machinery wherein m = number of sequences, *n* = total number of sites, S = Number of segregating sites, ps = S/n, T = ps/a1, *p* = nucleotide diversity, and D is the Tajima test statistic. **d** Tajima’s relative rate test however indicates that these species have evolved at different rates. All evolutionary analyses were conducted in MEGA6

In order to determine the evolutionary position of *C. auris* 6684 in the fungal genus tree, a concatenated phylogenetic tree was constructed based on orthologs of 95 conserved proteins (Additional file 2: Table S2) from 11 pathogenic species under the phylum *Ascomycota* (Fig. 3a). Our analysis shows bifurcation of *C. albicans* and *C. auris* 6684 in two distinct clades. However, we can see that *C. auris* 6684 and *C. lusitaniae* falls in the same clade, indicating convergence at the protein level. This is further confirmed by the amino acid substitution matrix of the house keeping machinery by maximum likelihood estimation wherein the number of amino acid substitutions per site between sequences is low (Fig. 3b). Tajima’s neutrality test indicates a positive D value which reflects low levels of polymorphism in the core housekeeping machinery of all these species including *C. auris* 6684 (Fig. 3c). Tajima’s relative rate test was performed to determine the heterogeneity of evolutionary rates between *C. lusitaniae* and *C. auris* 6684 with *C. albicans* used as an out group (Fig. 3d). The *χ*^2^ test statistic was 5.83 (*P* = 0.01580 with 1 degree of freedom). *P*-value was less than 0.05; hence null hypothesis was rejected, thereby indicating different rates of evolution for these species.

### *Candida auris* has a highly divergent genome

**Fig. 4:**
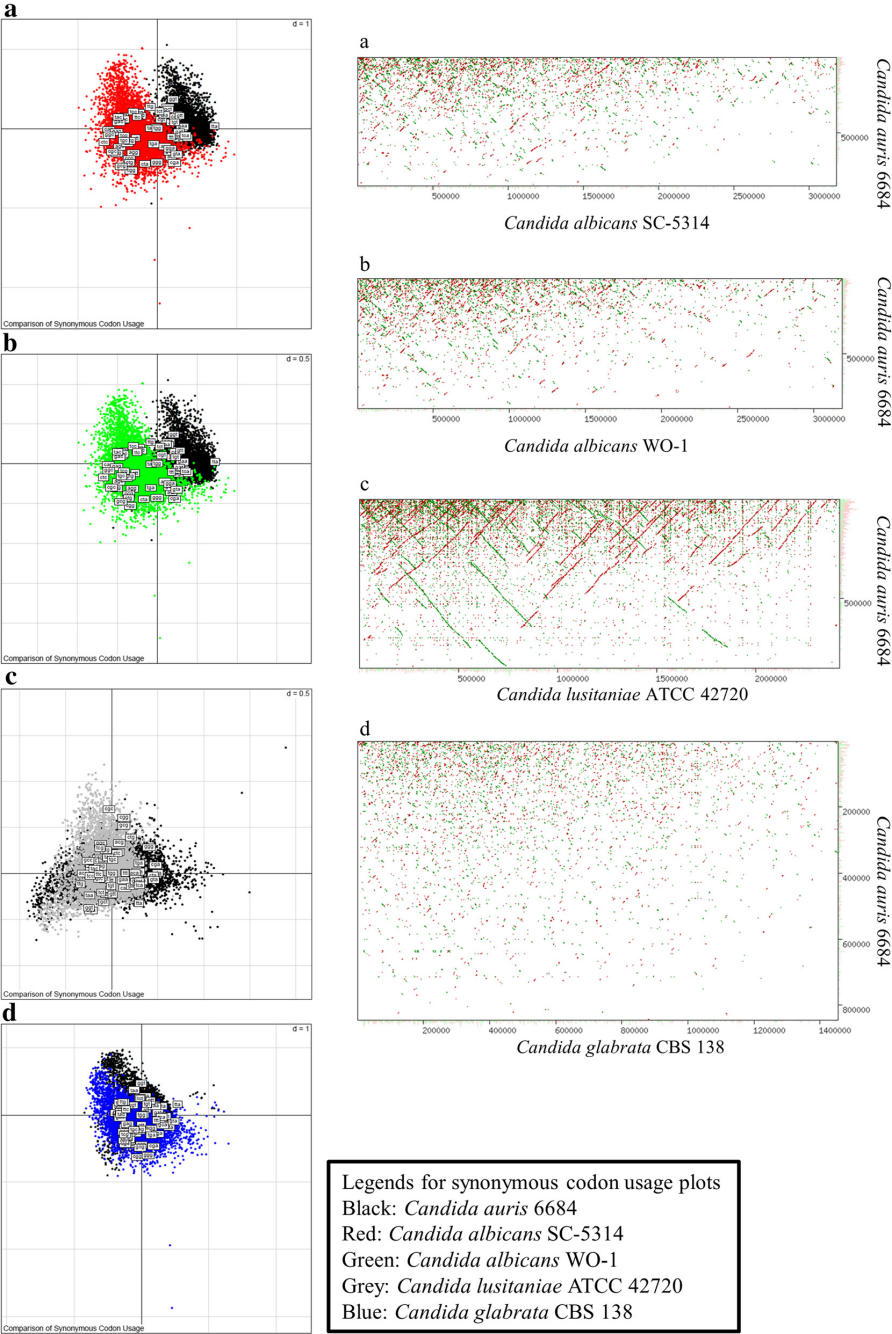
*Candida auris* has a highly divergent genome. **a**, **b**, **c**, **d** Synonymous Codon Usage distribution of *Candida auris* isolate 6684 with respect to *C. albicans* (SC-5314 (**a**) and WO-1 (**b**)), *C. lusitaniae* ATCC 42720 and *C. glabrata* CBS 138. These plots were generated by correspondence analysis and depict the variability in the sum of synonymous codon usage and amino acid usage. These graphs depict the codon usage bias relating it to the evolution of pathogenic fungus. **a**, **b**, **c**, **d** Whole (or draft) genome dot plot alignment showing genomic synteny of *Candida auris* isolate 6684 with respect to other well known pathogenic *Candida* species. The y-axis is the largest scaffold of *Candida auris* 6684 and the x-axis is the largest chromosome (or scaffold) of the corresponding genome being compared

**Fig. 5:**
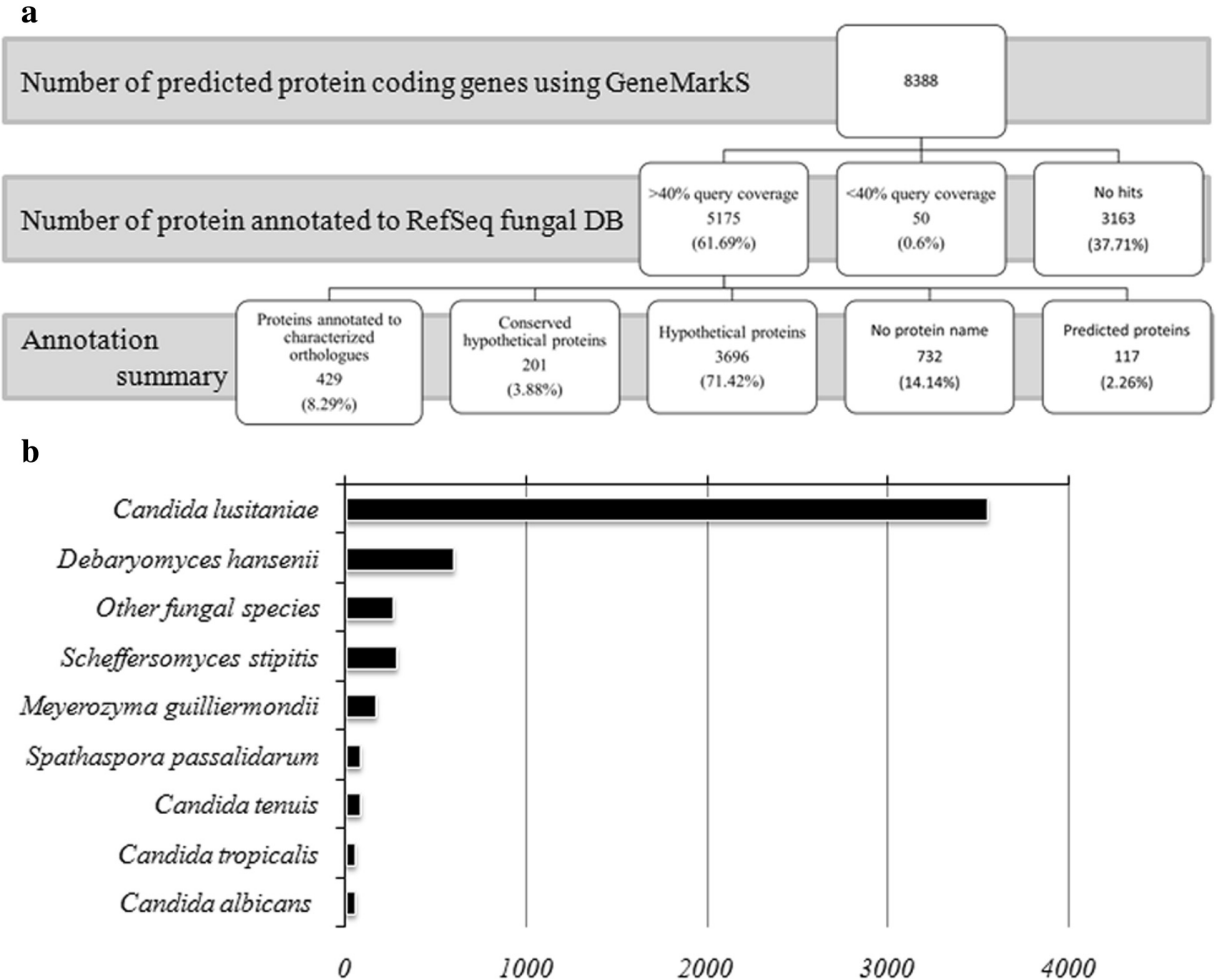
Summary of functional annotation of *Candida auris* genome. **a** Annotation results against RefSeq fungal protein database shows 5.1 % (429 out of 8358 protein coding genes) were annotated functionally with predicted names. The rest of the genome remains uncharacterized. **b** *C. auris* 6684 has highest number of orthologs in *C. lusitaniae* ATCC 42720. However most of them were annotated as hypothetical

To gain deeper insights into the genome conservation and evolution of *C. auris* with other pathogenic *Candida* species, we performed whole genome alignment of sequencing reads against *C. albicans* SC-5314*, C. glabrata* CBS-138*, C. lusitaniae* ATCC 42720 *and Saccharomyces cerevisiae* S288c. More than 99.5 % of the *C. auris* 6684 reads did not align to the current whole (or draft) genome sequences of these four species mentioned above*.* This indicates that *C. auris* 6684 is highly divergent at the genome level. To further investigate we compared synonymous codon usage between *C. auris* 6684, *C. albicans* SC-5314, *C. albicans* WO-1, *C. lusitaniae* ATCC 42720 and *C. glabrata* CBS-138 (Fig. 4a - d). The codon usage in *C. auris* 6684 shows very less overlaps to codon usage in *C. albicans* (SC-5314 and WO-1) as shown in Fig. 4a and b. The synonymous codon usage appears to be significantly overlapping for *C. auris* 6684 and *C. lusitaniae* which correlates and supports the relatedness found in the results of phylogenetic analyses (Figs. 3a and 4c). In addition, the codon usage in *C. auris* 6684 also shows fair overlap with *C. glabrata* where there was no similarity found at the genomic scale between the two (Fig. 4d). The difference in codon usage can be to enhance optimal protein structure and function from the already prevailing behaviours in *C. albicans*. This observation suggests the codon usage bias; which is required for understanding the selective pressures involved in evolution of these fungal species. In the same light, the dot plots of whole (or draft) genome comparison of *C. auris* 6684 with respect to *C. albicans* (SC-5314 and WO-1) and *C. glabrata* CBS-138 showed no linearity at the genome scale (Fig. 4a and d) which supports the observations seen in synonymous codon usage plots. *C. auris* 6684 genome seemed to have linear genomic synteny with *C. lusitaniae* genome which was very evident with the blastp results (Fig. 5) as well as synonymous codon usage.

In this study, genomic relatedness was carried out using GGD calculator (Genomic-to-Genomic Distance calculator), formula 2, performed at http://ggdc.dsmz.de (Meier-Kolthoff *et al.*, 2012). The GGD was calculated between *C. auris* 6684 and *C. albicans* (SC-5314 and WO-1), *C. lusitaniae* ATCC 42720, *C. glabrata* CBS-138 and *S. cerevisiae* S288c (Table 4). The genomic distances based on HSP/MUM (high-scoring segment pair/ maximal matches that are unique in both sequences) found out using BLAT [30] were on an average 0.20952, indicating the number of identical bases between the genomes is inversely proportional to the HSP length. The probability that these species belong to the same species or same subspecies is 0 as indicated by logistic regression of DNA-DNA hybridization (GGDC transform the genomic distances analogues to DNA-DNA hybridization).

**Table 4:** Genomic relatedness calculated using Genome-to-Genome Distance Calculator

Query genome	Reference genome	Formula 1 (HSP length/total length)				Formula 2 (identities/HSP length)				Formula 3 (identities/total length)			
		DDH	Model C.I.	Distance	Prob. DDH ≥ 70 %	DDH	Model C.I.	Distance	Prob. DDH ≥ 70 %	DDH	Model C.I.	Distance	Prob. DDH ≥ 70 %
*C. auris* 6684	*C. glabrata* CBS 138	8.4	2.29	0.957	0	20.3	2.29	0.2049	0	9.7	2.29	0.9653	0
*C. auris* 6684	*C. albicans* SC5314	8.8	2.34	0.934	0	19.8	2.34	0.2107	0	10	2.34	0.9473	0
*C. auris* 6684	*C. albicans* WO-1	8.8	2.33	0.934	0	19.8	2.33	0.211	0	10	2.33	0.9478	0
*C. auris* 6684	*C. lusitaniae* ATCC 42720	10.4	2.48	0.856	0	19.2	2.48	0.2171	0	11.4	2.48	0.8866	0
*C. auris* 6684	*S. cerevisiae* S288c	8.4	2.29	0.958	0	20.4	2.29	0.2039	0	9.6	2.29	0.9667	0

Distances are calculated by (i) comparing two genomes using the BLAT program to obtain HSPs/MUMs and (ii) inferring distances from the set of HSPs/MUMs using three distinct formulas. The distances are transformed to values analogous to DDH. The DDH estimate results from a generalized linear model (GLM) which also provides the estimate’s confidence interval (after the +/− sign). An additional bootstrap confidence interval is listed if this option was chosen in the job submission form. Logistic regression (with a special type of GLM) is used for reporting both the probabilities that DDH is > =70 % and > =79 %. GGDC is mainly used to calculate the *in silico* relatedness of the species

### Functional annotation of the *C. auris* 6684 genome

**Fig. 6:**
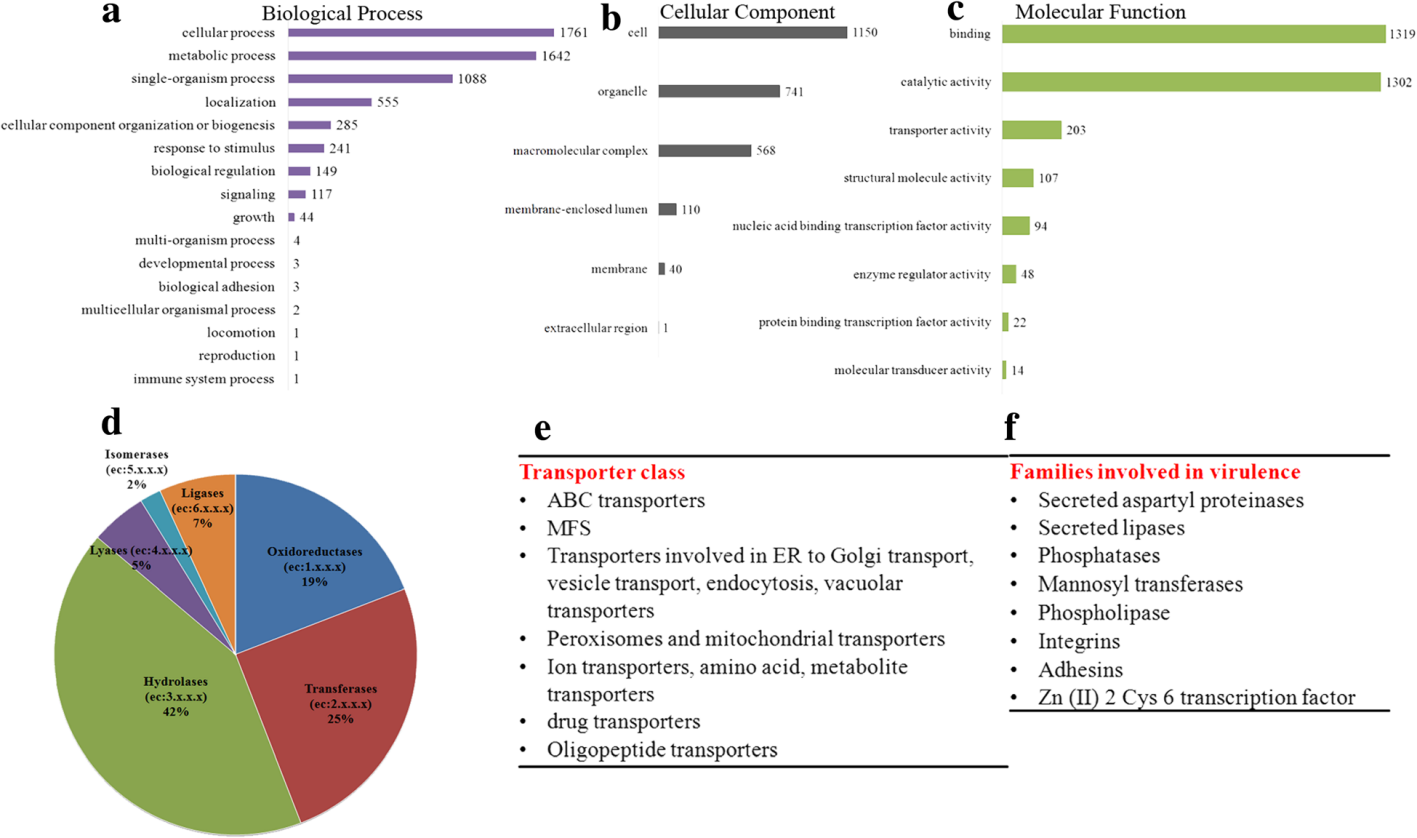
Functional annotation of *C. auris* genome. **a**, **b**, **c** Represents Level 2 GO terms for the three main domains. The most abundant terms in Biological process (**a**) is cellular process, metabolic process and single-organism process and in Molecular functions (**b**), binding, catalytic activity and transporter activity. The common cellular component (**c**) termed are the cell, organelle and membrane. **d** Distribution of various enzymes into the six enzyme classes according to E.C numbers. **e**, **f** represents gene families predicted in *C. auris* that are orthologous to *C. albicans*

Functional annotation was done in Blast2GO that combined the blastp annotation results (against NR database) with the predicted InterProScan results. The assigned GO descriptions to each protein were considered at an E-value greater than e^−10^. Out of 8358 predicted proteins 10958 GO terms were annotated to 3560 sequences. The GO terms were placed in three domains, Biological process (39.45 %), Molecular Function (43.25 %) and Cellular Components (16.52 %). Figure 6a-c represents the level 2 GO terms for all the three domains. As evident from Fig. 6a, a major proportion of the genome is devoted to cellular and metabolic processes. A significant number of proteins were annotated to have transporter activity apart from binding and catalytic activity.

We also performed enzyme classification analysis based on Enzyme Commission (EC) numbers predictions for each sequence. We found that hydrolases are the largest group of *C. auris* 6684 enzymes (42 %), followed by transferases (25 %) and oxidoreductases (19 %). Blast2GO identified 466 enzyme (Fig. 6d) out of which 329 enzymes got mapped to KEGG pathways. BlastKOALA was used to reconstruct KEGG pathways for *C. auris* 6684. 2775 proteins (out of 8358 predicted proteins) got annotated into various pathways. This analysis revealed that the central pathways pertaining to carbohydrate, lipid and amino acid metabolisms are conserved.

### Core circuitry related to virulence is conserved in *C. auris* 6684

Considering the high genomic variability of *C. auris* 6684, we asked the question that whether gene families that are known to have a role in pathogenicity of *Candida* species [31] are also conserved in *C. auris* 6684*?* We used the genome of *C. albicans* SC5314 as the template gene model to predict orthologs in our isolate as it is well annotated at the experimental level. This approach yielded 1988 orthologous proteins with functional annotations. Our analysis predicted an arsenal of transporters orthologous to that of *C. albicans*, belonging primarily to the major facilitator superfamily and ABC (ATP binding cassette) superfamily [32] (Fig. 6d). The up regulation of these multidrug efflux pumps may explain the intrinsically low susceptibility of *C. auris* 6684 to antifungal drugs. Apart from the general transcription factors, 193 proteins were predicted to have DNA binding/sequence specific DNA binding/transcription factor activity. We also predicted a multitude of zinc finger transcription factors orthologous to those present in *Saccharomyces cerevisiae, Candida albicans* and *Scheffersomyces stipites.* Notably the Zn (II) 2 Cys 6 transcription factor family is enriched in our isolate (26 in number). Four of these are known to be key regulators of MDR1 transcription in *C. albicans*; gain-of-function mutations of which leads to up regulation of multidrug efflux pump MDR1, thereby leading to multidrug resistance [32, 33].

The genome was found to contain transcription factors like STE-related and MADS-box proteins which have been previously shown to be involved in the virulence of human fungal pathogens [34, 35] and plant fungal pathogens [36, 37] respectively. Ste12p is conserved in many fungi, regulating processes involved in mating, filamentation, substrate invasion, cell wall integrity and virulence [34], while MADS-box proteins bind to DNA and have dimerization activity [35]. Our analysis also indicated conservation of the Rim101 transcriptional pathway that is known to respond to alkaline pH in *Saccharomyces cerevisiae*. 122 proteins were predicted to have kinase/phosphorylation activity. Out of this, 93 proteins have the serine/threonine kinase domain and the rest were predicted to be involved in protein phosphorylation due to the presence of putative kinase domain/ATP binding domain. *C. auris* 6684 draft genome encodes for kinases like Hog1, Protein Kinase A (PKA) and two-component histidine kinase. Activation of stress signaling pathways regulated by these protein kinases have been implicated to enhance tolerance of pathogenic fungi to chemical fungicides and antifungal peptides [38]. HOG1 protein is a fungal mitogen-activator protein (MAP) kinase which has been implicated in responses to oxidative and hyperosmotic stresses in a few human pathogens including *C. albicans* [39]. PKA is shown to be activated in response to extracellular nutrients and subsequently regulates metabolism and growth, while two-component histidine kinase is shown to be critical to morphogenesis and virulence [31, 40, 41].

We also identified eight *OPT* genes encoding putative oligopeptide transporters which have been implicated in the acquisition of nutrient versatility thereby helping the pathogen to adapt to various host niches [42]. Interestingly it has been reported that in *C. albicans*, these genes are also induced upon phagocytosis by macrophages [43]. We also found orthologs of genes predicted to be hexose transporters, maltose transporters and permeases (amino acid permeases, sulfur permeases, allantoate permeases, glycerol permeases and iron permeases) which further expands its nutrient assimilation machinery, thereby helping it to acclimatize to diverse host niches.

Our next step was to hunt down the attributes that may explain the aggressive behavior of the pathogen. Our analysis indeed predicted many known virulence associated genes (Fig. 6e). Since the cell wall serves as the interface between the pathogen and the host immune defense, components of the cell wall serve as pathogen associated molecular patterns and virulence factors. Our analysis indicated that the family of mannosyl transferases is conserved in *C. auris* 6684 with many predicted orthologs. Apart from maintaining cell wall architecture by coordinating glycan synthesis, these enzymes play a very important role in immune recognition, host cell adherence and virulence in *C. albicans* [44]*.* Integrins and adhesins are the other two gene families which have a crucial role in adherence and virulence of *C. albicans* [45, 46]. However our annotation predicted only two proteins, one structurally similar to alpha-subunit of human leukocyte integrin; predicted to play a role in morphogenesis, adhesion, mouse coecal colonization and virulence; and another secreted protein similar to alpha agglutinin anchor subunit which has been previously shown to be induced upon exposure to fluconazole. This clearly suggests that *C. auris* employs distinct mechanisms for host cell adhesion.

We also found four orthologs of secreted aspartyl proteases (SAP) two of which were predicted to have greater expression upon deep epidermal invasion; greater expression in vaginal than oral infection [47] and prominent role in biofilm formation. We also found two genes annotated as vacuolar aspartic proteinases. The secreted aspartic proteinases help the fungus to digest host proteins and the resulting peptides are taken up into the cell by specific transporters like the oligopeptide transporters family mentioned above [48]. Our results also annotated eight genes orthologous to secreted lipases. In all, our analysis revealed that enzyme families implicated in invasiveness like mannosyl transferases, secreted aspartyl proteases and lipases are enriched in our clinical isolate. However the adhesion and integrin gene families are ill represented. This information has been categorized in Additional file 2: Table S3. Our analysis also revealed 686 proteins predicted to be induced or repressed upon rat catheter or biofilm formation. This includes a multitude of enzymes, transcription factors, ribosomal proteins and transporters. This clearly indicates that *C. auris* 6684 has significant ability to form biofilms since the core genes involved in biofilm formation are conserved. However experiments need to be done to validate the same.

### Structure of mating loci in *C. auris* and PCR based diagnostic test to differentiate between *C. auris* and *C. haemulonii*

Another peculiarity seen in *Candida* species is the highly diverse nature of sexuality. Diploids like *C. tropicalis* and *C. parapsilosis* are unable to mate while *C. albicans* shows a parasexual cycle. Haploids like *C. lusitaniae* and *C. gulliermondii* are heterothaliic in nature [49]. It is interesting to note that virulence and mode of reproduction are being analysed as linked phenomenon in recent years. *C. lusitaniae* is a heterothallic species known to be involved in sexual reproduction. On the other hand certain *Candida* species are either parasexual or asexual. Considering the high similarity shared by *C. auris* 6684 and *C. lusitaniae*, we speculated that *C. auris* 6684 might have a sexual stage similar to the latter. Sexual mating is controlled by a single genetic locus called the MAT locus consisting of two alleles-MATa and MATα.

**Fig. 7:**
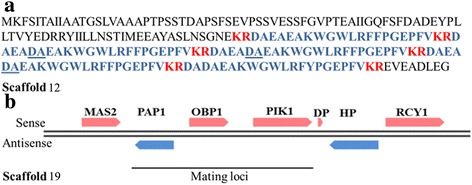
Identification of mating gene MF α and mating loci in *C. auris.* **a** The amino acid sequence of the MF α gene. Regions encoding the mature α pheromone peptide is shown in blue color. Possible Kex2 cleavage sites are shown in red colour. Additional DA residues present in three of the peptides have been underlined. **b** Scaffold 18 shows conservation of non sex genes of MTL locus, however, MF α gene is present in a different scaffold

To understand the mode of reproduction in *C auris*, we analysed the MAT loci (*MTL*) in the genome assembly. Our search led to the identification of a putative gene sequence in *C. auris* 6684 genome with similarities to α mating pheromone of *Naumovozyma castellii* CBS 4309*.* The gene sequence consists of a 654-bp ORF that encodes for five putative α pheromone peptide repeats separated by KEX2 proteinase cleavage sites. Two of the five α-peptides are identical in sequence; the remaining three contains additional DA residues (Fig. 7). We also found a homologue of KEX2 in the genome. However, the genes in the vicinity of MF-α were all annotated as hypothetical (Additional file 2: Table S4). Interestingly, the three non-sex genes (NSGs) of the *MTL* locus namely, the essential phosphatidyl inositol kinase gene (*PIK)*, the essential poly (A) polymerase gene (*PAP)*, and the nonessential oxysterol binding protein gene (*OBP)* were present in a different scaffold (Fig. 7). In *C. albicans*, these genes have been implicated in biofilm impermeabilty and fluconazole resistance [50]. Thus MAT α gene is located in a different locus. In *C. auris* 6684, *ERG11* is also located on the same scaffold as *MTL* non sex genes and in *C. albicans*, the loss of heterozygosity at the *MTL* locus has been correlated to azole resistance [51]. However we could not find *MAT*a gene in the genome. Thorough experimentation needs to be done to establish its sexuality.

**Fig. 8:**
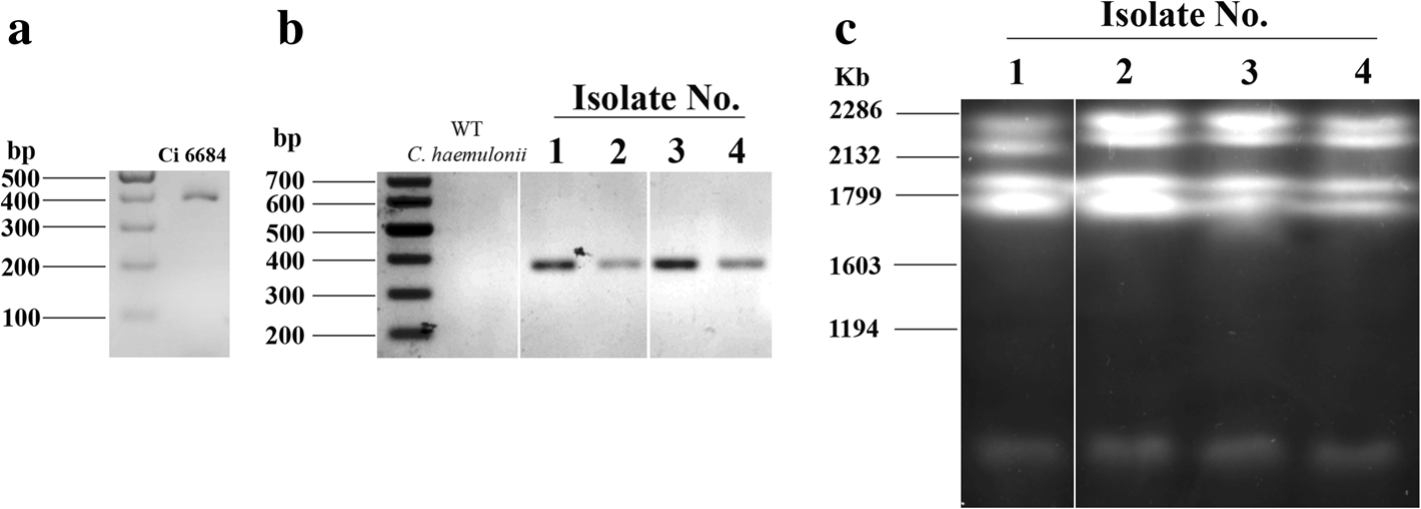
PCR based diagnostic method to differentiate between *C. auris* and *C. haemulonii*. **a** Primers based on *C. auris* 6684 MFα gene gives a specific amplicon at 400 bp. **b** Amplification was not seen in the case of *C. haemulonii* 8176. However, four clinical isolates identified to be *C. haemulonii* by Manipal Hospital, showed a band at 400 bp. **c** The PFGE profile of these four clinical isolates was similar to that of *C. auris* 6684

The sequence of the gene coding mating factor α is unique to each *Candida* species and therefore we designed PCR primers specifically for *MF* α gene. This PCR was tested on *C. haemulonii* 8176 obtained from MTCC, IMTECH and *C. auris* 6684. As evident in Fig. 8a, *C. auris* 6684 gave an amplicon at 400 bp which was not seen for *C. haemulonii*. This test was further extrapolated to other clinical isolates reported to be *C. haemulonii* and many of them turned to be PCR positive for *C. auris* (Fig. 8b). The same isolates also showed a similar PFGE pattern (Fig. 8c), thereby confirming the fact that these were misdiagnosed as *C. haemulonii.*

## Conclusions

Opportunistic infections caused by *Candida* are on the rise globally and newer pathogenic species are emanating at an unprecedented rate. What are not evolving at the same pace are the current methods of diagnosis and treatment options leading to misdiagnosis and clinical failure. The last decade has witnessed the emergence of newer species called *C. haemulonii* from being the causative agent of minor infections to one of the leading causes of invasive infections. It is currently increasing in prevalence, with several ongoing outbreaks in developing and underdeveloped countries. The actual incidence rate is however misleading because of the inability of the current automated systems used for screening of fungal species to identify novel emerging fungal pathogens such as *C. auris*, *C. pseudohaemulonii* and other related species due to striking similarities in biochemical characters and the unavailability of molecular markers for accurate identification. We have generated the first draft genome sequence of a commonly misdiagnosed, emerging pathogen *C. auris*. The isolate was identified as *C. haemulonii* by Vitek2. However PFGE analysis revealed 5 bands similar to that of *C. auris* and accurate species identification was done by phylogenetic analysis based on the partial sequence of 18S rRNA, ITS1, 5.8S rRNA complete sequence, ITS2 and 28S rRNA partial sequence. Genome sequencing will highlight important differences which may act as accurate identification markers for this group of emerging pathogens at the species level. Towards this we have developed a PCR based diagnostic test to distinguish between these two pathogens.

The genome of *C. auris* spans about ~12.5 Mb with 8358 predicted protein coding genes. Strikingly, at the genomic level, *C. auris* shows a highly divergent relationship with other pathogenic *Candida* species as indicated by a meagre 0.5 % alignment of the sequencing reads to other *Candida* genomes and supported by lack of linear synteny of genomic dot plots. *C. auris* is phylogenetically closest to *C. haemulonii* whose genome sequence is unavailable. Among the sequenced yeast species, it is closest to *C. lusitaniae*; however, its genome is also not well annotated functionally. Therefore majority of the protein coding genes were predicted to be hypothetical/functionally uncharacterized. The role of each of these unique candidate proteins demands for urgent functional studies. Hence accurate identification and *de novo* assembly and annotation still remains a challenge for divergent sequences among emerging pathogenic species. 37.71 % of the protein coding genes showed no sequence similarity to genes available in public database, thus indicating that speciation genes are embedded within the genome which may be involved in grooming it as an aggressive pathogen. With the limited data available, it is difficult to comment about the genomic architecture of speciation and how it facilitates or impedes further divergence. To further probe into the difference at the functional level we resorted to synonymous codon usage plots which distinguish ways by which translational selection of protein coding genes occurs among related species. The above observation is supported by GGDC that calculated the *in silico* relatedness of *C. auris* and sequenced *Candida* pathogens, surprisingly the logistic regression quantifies no relatedness among the species. The ecological niche of most of these *Candida* species is known, that may throw light on the evolutionary forces grooming these organisms at the species level. However till date there are no reports of naturally occurring *C. auris* species. *C. auris* can grow at elevated temperatures of 42 °C whereas *C. haemulonii* cannot. This gives us a hint that *C. auris* has the potential to infect the avian fauna whose body temperature is in the range of 40 °C to 42 °C. However, additional experiments need to be done in order to validate this phenomenon.

The foremost criterion to be a successful *Candida* pathogen is the ability to colonize diverse anatomical niches within the host such as skin, oral cavity, gastrointestinal tract, vagina and the vasculature. Each *Candida* pathogen has its own machinery dedicated to host cell adhesion, recognition, invasion and colonization. We compared *C. auris* genome with that of *C. albicans* since it is well annotated and well-studied as well as distantly related to *C. auris*. While the spectrum of virulence traits like hyphae formation, white opaque switching is quite different between these two species, we found that *C. auris* still shares some common virulence traits with *C. albicans*. Our analysis highlights that a significant portion of *C. auris* genome encodes for transporters belonging to the ABC transporter family and major facilitator superfamily. This may partly explain its increased tolerance to antifungal drugs. The multidrug resistant nature of the pathogen and the limited arsenal of antifungal agents indicate that there is a critical need for finding new drug targets and genome sequence of *C. auris* therefore may prove useful in finding alternative targets that can augment the existing antifungal therapy. Our analysis also provides a snapshot of the potential genetic attributes that may explain its virulent nature. The genome of the pathogen harbours gene families such as lipases, oligopeptide transporters, mannosyl transferases and transcription factors which play a multitude of roles in colonization, invasion and iron acquisition. Also majority of genes known to be involved in formation of biofilm appears to be conserved. In all, we see that *C. auris* shares many genes with *C. albicans* and *C. lusitaniae* indicating a common ancestry; however it may have acquired novel genetic traits that have groomed it as a specialist pathogen. It is possible that the indiscriminate use of antibiotics shaped its genome to expand not only its clinical spectrum of infection but also to emerge as a successful multidrug resistant pathogen.

In all, our study provides the first whole genomic overview of *C. auris*, the first member of the *Candida haemulonii* and related pathogenic fungi complex to be sequenced. This report is a major step toward the initiation of genomic studies of this complex group of fungi which are fast turning drug resistant and may be a menace with limited treatment options available in the future.

## Methods

### Strain and growth conditions

All clinical isolates were obtained from Manipal Hospital, Bengaluru and the ethical approval was obtained from Ethics Committee of Manipal Hospitals, Bengaluru and informed consent was taken as required during the study. Ci 6684 was isolated from a patient who had sepsis with multiorgan dysfunction. *C. haemulonii* 8176 was obtained from MTCC, IMTECH Chandigarh, India. Strains were routinely grown in Yeast Peptone Dextrose (YPD) medium at 37 °C.

### Minimum inhibitory concentration and growth assays

To determine the *in vitro* susceptibility to antifungal drugs, broth microdilution protocol [52] was used. Overnight cultures were grown at 37 °C in YPD. Approximately 10^3^ cells per well in YPD media at 37 °C. Minimum inhibitory concentration (MIC) tests were set up in a total volume of 0.2 ml/well with 2-fold dilutions of drugs. Fluconazole gradients where in the following concentration steps in μg/ml: 64, 32, 16, 8, 4, 2, 1, 0.5, 0.25, 0.125, 0.0625 and 0.03125. For Amphotericin B, gradients where in the following the concentration steps in μg/ml were: 16, 8, 4, 2, 1, 0.5, 0.25, 0.125, 0.0625, 0.03125 and 0.015625. 24 or 48 h post incubation, growth was measured by reading the optical density at 600 nm after agitation using a spectrophotometer (Tecan). MIC_50_ was defined as the concentration of drug reducing growth by 50 % relative to the wells containing no drug. Sterile water was the vehicle for Fcz and AmB.

### DNA sequencing

Short reads and long reads library preparation was performed at Genotypic Technology’s Genomics facility following NEXTFlex DNA library protocol outlined in “NEXTFlex DNA sample preparation guide (Cat # 5140–02). ~3 μg of genomic DNA was sonicated using Bioruptorto and 300 to 600 bp sized fragments were obtained. The size distribution was checked by running an aliquot of the sample on Agilent HS DNA Chip. The resulting fragmented DNA was cleaned up using Agencourt AMPure XP SPRI beads (Beckman Coulter). Fragmented DNA was subjected to a series of enzymatic reactions that repair frayed ends, phosphorylate the fragments, and add a single nucleotide A overhang and ligate adaptors (NEXTFlex DNA Sequencing kit). Sample cleanup was done using AMPure SPRI beads. After ligation-cleanup, ~300–600 bp fragments was size selected on 2 % low melting agarose gel and cleaned using MinElute column (QIAGEN). PCR (10 cycles) amplification of adaptor ligated fragments was done and cleaned up using AMPure SPRI beads. The prepared libraries were quantified using Qubit flourometer and validated for quality by running an aliquot on High Sensitivity Bioanalyzer Chip (Agilent). The short read inserts were sequenced in Illumina MiSeq and long read inserts were sequenced in Illumina NextSeq 500.

Mate-pair reads library preparation was performed at Genotypic Technology’s Genomics facility following Nextera Mate Pair Gel Plus protocol outlined in “Illumina Nextera Mate Pair library preparation guide (Cat# FC-132-9001DOC, Part#15035209 Rev D.)”. ~4 μg of Qubit quantified DNA was taken for Tagmentation. The tagmented sample was cleaned up using AMPure beads and subjected to strand displacement. 3–5 kb range of the strand displaced sample was size selected on 0.6 % agarose gel. Size selected sample was taken for circularization overnight, followed by linear DNA digestion with DNA Exonuclease. The circularized DNA molecules were sheared using Covaris to obtain fragments in the size range of 300 to 1000 bp. Sheared DNA was subjected to bead binding with M280 Streptavidin beads to isolate biotinylated molecules. End repair, A-Tailing and adapter ligations were performed on the bead-DNA complex. Adaptor ligated sample was amplified for 15 cycles of PCR followed by AMPure XP bead clean up. The prepared library was quantified using Qubit and validated for quality by running an aliquot on High Sensitivity Bioanalyzer Chip (Agilent). The mate-pair reads were sequenced using Illumina NextSeq 500.

### Assembly, annotation and analysis

The qualities of the reads were checked using Genotypic proprietary tool SeqQC v2.21. The average sequencing depth (coverage) for short paired-end reads is 158.19x, long paired-end reads is 175.51x and mate-pair reads is 205.78x. Processed short paired-end reads (3.27 million) were used to generate (250–400) long fragments using ARF-PE v0.2. 467178 long fragments were generated using 467178*2 paired end reads (ie, 14.29 % reads were used in long read generation). 467178 long fragments and 3269025*2 paired end reads used for Newbler Genome assembly. Newbler version 2.8’s default assembly parameters were used for the assembly and 721 scaffolds were generated. The paired-end long insert reads and mate-pair reads were used to gap fill using SSPACE-STANDARD v3.0 [53] and the contigs were reduced to 65 scaffolds. Using Reapr v1.0.17 [54], the 65 scaffolds were corrected, removing the erroneous bases and the final number of scaffolds was 97. These 97 scaffolds were used as input for GeneMarkS [55] to predict protein-coding genes with –eukaryotic as the main option. The resulting 8388 proteins were subjected to local blastp, resulting in 5175 proteins being annotated to RefSeq fungal protein database. Proteins having query coverage of greater than 40 % were only considered from this blast results. An InterproScan [56] was carried out using the tool Blast2GO [57] v3.0 to group the predicted proteins according to the presence of domain/motif in their sequences. GO terms were assigned through Blast2GO tool based on NR Database orthologs (blastp with Evalu > e^−10^). Proteins involved in various KEGG pathways were assigned using BlastKOALA [58]. Transfer RNAs were identified using the tRNAScan-SE program [59]. Ribozomal RNAs were predicted by RNAmmer [60]. The sequenced reads were mapped to various pathogenic *Candida* genome using Bowtie2 v2.2.3 [61] with default parameter. The generated SAM files were used to calculate the percent of reads aligned using R.

### Electrophoretic karyotyping

Modified PFGE, Counter-clamped homogeneous electrical field (CHEF) (BIO-RAD) was used for electrophoretic karyotyping of *C. auris* 6684 and *C. albicans*. The protocol was adapted from Iadonato *et al.* 1996 [62]. Briefly 5 ml yeast cultures were grown in YPD medium at 30 °C. The cells were the harvested and washed with 50 mM EDTA. Approximately 2× 10^9^ cells/ml were added to equal volumes of 1 % (w/v) low melt Pulse Field certified Agarose (BIO-RAD), prewarmed at 45 °C. The mixture was then transferred in to disposable plug moulds to harden. Plugs were then extruded and suspended in freshly prepared spheroplasting solution containing Zymolase, and incubated at 37 °C for 4 h. After this the plugs were washed with 1 % Lithium dodecyl sulfate (LDS) (2X 30 min) buffer followed by cell lysis with 1 % N-lauryl sarcosine (NDS) (3X 30 min) buffer. Finally the plugs were rinsed (6x 30 min) with TE buffer pH 8. Agarose plugs containing yeast DNA was then loaded into 0.8 % low melt Pulse Field certified Agarose (BIO-RAD) prepared with 0.5X TBE buffer. The DNA samples were resolved by running the gel in CHEF-DR® III system with 5 V/cm^2^ with pulse time of 120 s and total run time of 36 h at 12 °C. Gel was then stained with ethidium bromide (1ug/ml) for 30 min and visualized at ImageQuant LAS 4000 transilluminator (GE).

### Phylogenetic tree and evolutionary analysis

The partial sequence of 18 s rRNA, ITS1, 5.8 s rRNA complete sequence, ITS2 and 28 s rRNA partial sequence retrieved from NCBI (Additional file 2: Table S5) were used to categorise Clinical isolate 6684 with *Candida auris* clade. The evolutionary tree was inferred using the Maximum Likelihood method based on the Tamura-Nei model [63]. The tree with the highest log likelihood (−307.3435) is shown. The percentage of trees in which the associated taxa clustered together is shown next to the branches. Initial tree(s) for the heuristic search were obtained automatically by applying Neighbor-Join and BioNJ algorithms to a matrix of pairwise distances estimated using the Maximum Composite Likelihood (MCL) approach, and then selecting the topology with superior log likelihood value. The tree is drawn to scale, with branch lengths measured in the number of substitutions per site. The analysis involved 48 nucleotide sequences. All positions containing gaps and missing data were eliminated. There were a total of 167 positions in the final dataset.

95 conserved proteins (Additional file 2: Table S2) from *Saccharomyces cerevisiae* S288c were retrieved using YGD, CGD and BLASTn for the following organisms: *Saccharomyces cerevisiae* S288c*, Candida albicans* SC-5314*, Candida dubliniensis* CD-36*, Candida glabrata* CBS 138*,,Candida* isolate 6684*, Candida tropicalis* MYA-3404*, Candida lusitaniae* ATCC 42720*, Candida gulliermondii* ATCC 6260*, Candida orthopsilosis* Co-90–125*, Ashbya_gossypii* and *Histoplasma capsulatum.* The phylogenetic tree was constructed using the Neighbor-Joining method. The optimal tree with the sum of branch length = 1.22757517 is shown. The percentage of replicate trees in which the associated taxa clustered together in the bootstrap test (2000 replicates) is shown next to the branches. The tree is drawn to scale, with branch lengths in the same units as those of the evolutionary distances used to infer the phylogenetic tree. The evolutionary distances were computed using the *p*-distance method and are in the units of the number of amino acid differences per site. The analysis involved 11 amino acid sequences. All positions with less than 95 % site coverage were eliminated. There were a total of 51712 positions in the final dataset.

Tajima’s neutrality analysis involved concatenated amino acid sequences from the 11 species. All positions with less than 95 % site coverage were eliminated. There were a total of 51712 positions in the final dataset. The equality of evolutionary rate between *Candida lusitaniae*, Clinical isolate 6684 with *Candida albicans* as an out-group was determined by Tajima’s relative rate test [64, 65]. All positions containing gaps and missing data were eliminated. There were a total of 56989 positions in the final dataset. All the phylogenetic trees and evolutionary analyses were conducted in MEGA6 [66] .

### Genome comparison

For genome comparison the current genome sequences (whole or draft) were downloaded from Broad Institute (https://www.broadinstitute.org/scientific-community/science/projects/fungal-genome-initiative/fungal-genomics) and CGD (www.candidagenome.org/). The analysis was carried out using GFFex v2.3 and Biostrings package of Bioconductor in R v3.1. The DNA-DNA hybridizations (DDH) distances were calculated using the online tool Genome-to-Genome Distance Calculator (GGDC 2.0) (http://ggdc.dsmz.de/). Dot plot were done in an online tool called YASS [67] by setting the e-value to e-10 and the synonymous codon usage plots were done in R (v3.1) using ape4 and seqinr packages [68] of Bioconductor.

### Polymerase chain reaction

Genomic DNA was isolated as described previously. Based on the MFα region sequence from *C. auris*, a specific PCR-based method was developed for the direct detection of *C. auris* DNA by using a *C. auris* -specific primer (CaMF [5′- GAGAAAAGAGACGCTGAAGCTGAG-3′]) designed using the gene sequence which codes for the unique pheromone together with reverse primer (CaMR [5′- TCAACCTTCGAGGTCAGCTTCA-3′]).

### Ploidy analysis by FACS

Cultures were grown in YPD till A600 of 1.0. The cells were washed in 1X PBS (137 mM NaCl, 2.7 mM KCl, 10 mM sodium phosphate dibasic (NaH2PO4), 2 mM potassium phosphate monobasic (K2HPO4), pH of 7.4) and fixed in 70 % ethanol for 1 h at room temperature or kept at 4 °C overnight. The cells were suspended in 1X PBS and incubated with RNase A (1 mg/ml) at 37 °C for 4 h in the same buffer. Cells were subsequently washed with PBS, and finally stained with propidium iodide (PI, 16 μg/ml) for flow cytometric analysis in BD FACS Canto.

### Availability of supporting data

The whole genome sequencing data can be accessed through BioProject accession number PRJNA267757. The respective BioSample accession numbers is SAMN03200169. The SRA reference numbers of the whole genome sequencing are SRX766223 (Illumina MiSeq short paired-end reads), SRX766234 (Illumina NextSeq 500 mate-pair reads) and SRX766231 (Illumina HiSeq2500 long paired-end reads). This Whole Genome Shotgun project has been deposited at DDBJ/EMBL/GenBank under the accession LGST00000000. The version described in this paper is version LGST01000000.

## Additional files

Additional file 1:
**Figure S2.** Colony morphology of *C. auris* and *C. albicans* SC-5314. (PNG 147 kb) Additional file 2:
**Table S1.** Number of reports regarding various *Candida* infections. **Table S2:** 95 Conserved Proteins in *Candida* and *Saccharomyces* used for phylogenetic analysis. **Table S3:** Related Protein Families as categorized based on orthologues to *C. albicans*. **Table S4:** Upstream and Downstream Neighbours of MAT alpha loci. **Table S5:** NCBI accession numbers used for Phylogenetic analysis. (XLSX 26 kb) Additional file 3:
**Figure S1.** Pipeline depicting the methods used for *de novo* assembly and functional annotation of *C. auris* 6684 (or Ci 6684) draft genome. (TIFF 6662 kb) Additional file 4:
**Figure S3.** Flow cytometric analysis of DNA content of *Candida* species. (JPEG 76 kb) Additional file 5:
**Multiple sequence alignment of rRNA and ITS sequences of** ***C. auris*** **6684 and reported Indian strains of** ***C. auris*** **from NCBI.** (PDF 122 kb)

## Abbreviations

MDR: Multidrug resistance
ITS: Internal transcribed spacer
HAI: Hospital acquired infections
AmB: Amphotericin B
Fcz: Fluconazole
MIC: Minimum inhibitory concentration
PFGE: Pulse field gel electrophoresis
GGDC: Genomic to genomic distance calculator
GO: Gene ontology
KEGG: Kyoto encyclopedia of genes and genomes
ABC: ATP binding cassette
OPT: Oligopeptide transporters
SAP: Secreted aspartyl proteinases
MTL: Mating locus
