# Supplementary figures and images for: Draft genome of a commonly misdiagnosed multidrug resistant pathogen Candida auris
Source: BMC Genomics. 2015 Sep 7;16(1):686. doi: 10.1186/s12864-015-1863-z (PMC4562351; doi:10.1186/s12864-015-1863-z)

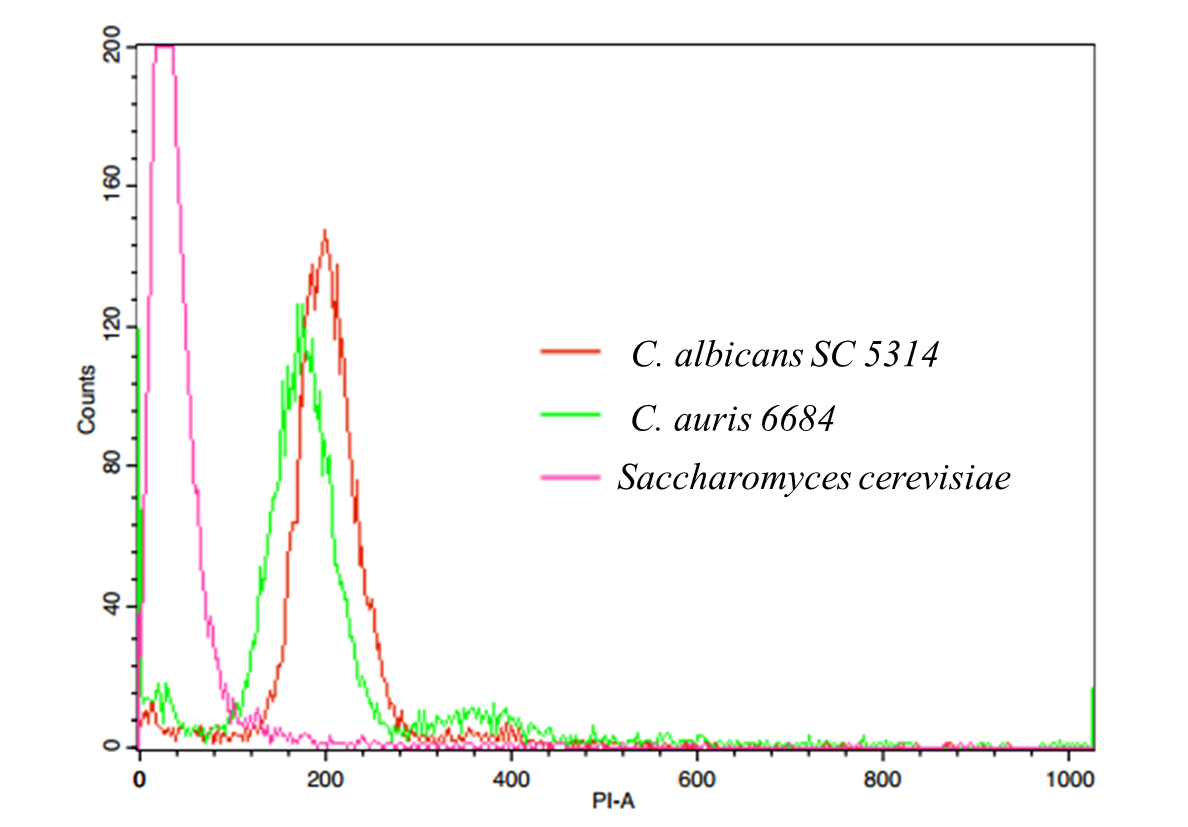

Supplement: Additional file 1: — Figure S2. Colony morphology of C. auris and C. albicans SC-5314. (PNG 147 kb) [file 12864_2015_1863_MOESM1_ESM.png]

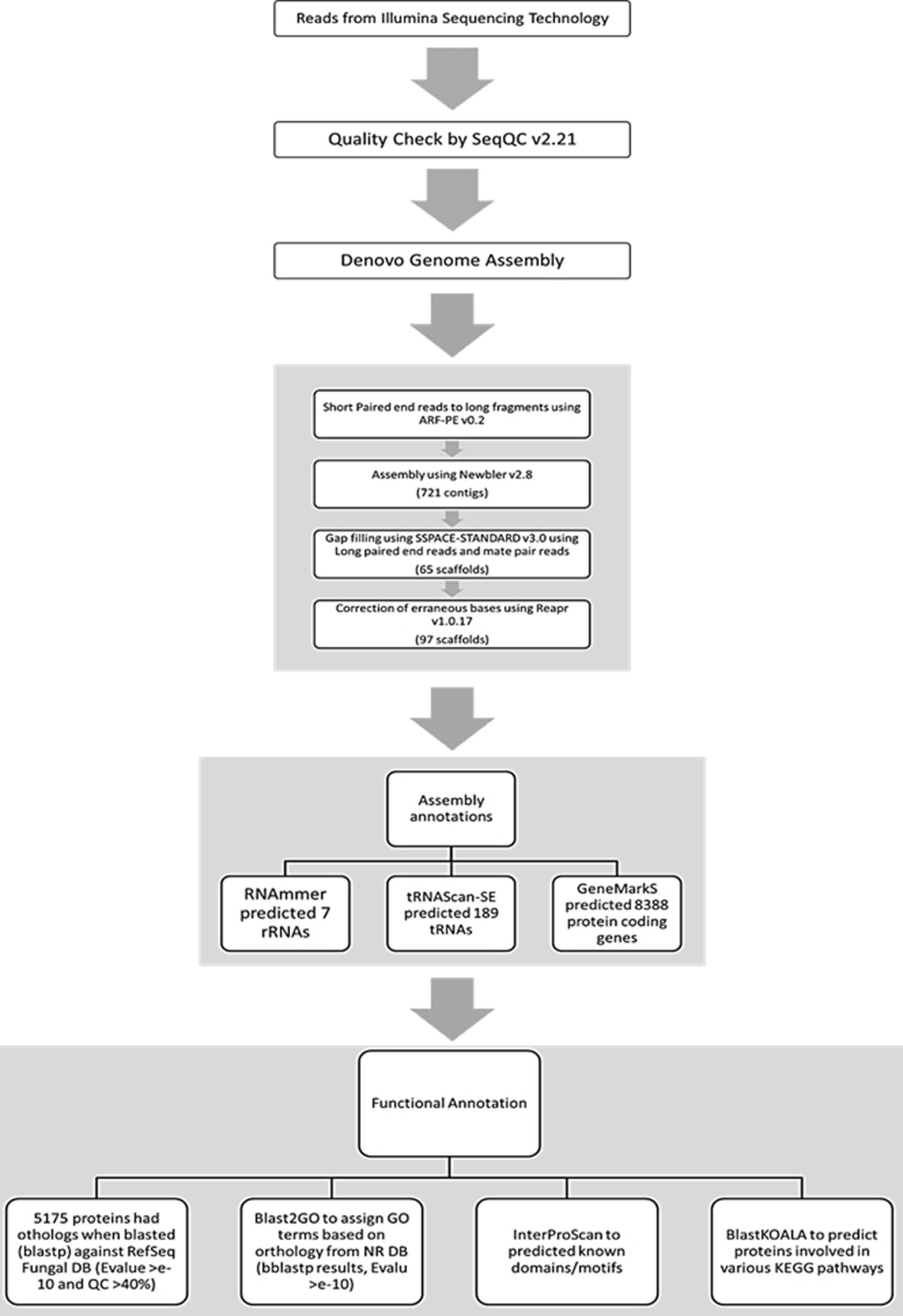

Supplement: Additional file 3: — Figure S1. Pipeline depicting the methods used for de novo assembly and functional annotation of C. auris 6684 (or Ci 6684) draft genome. (TIFF 6662 kb) [file 12864_2015_1863_MOESM3_ESM.tiff]

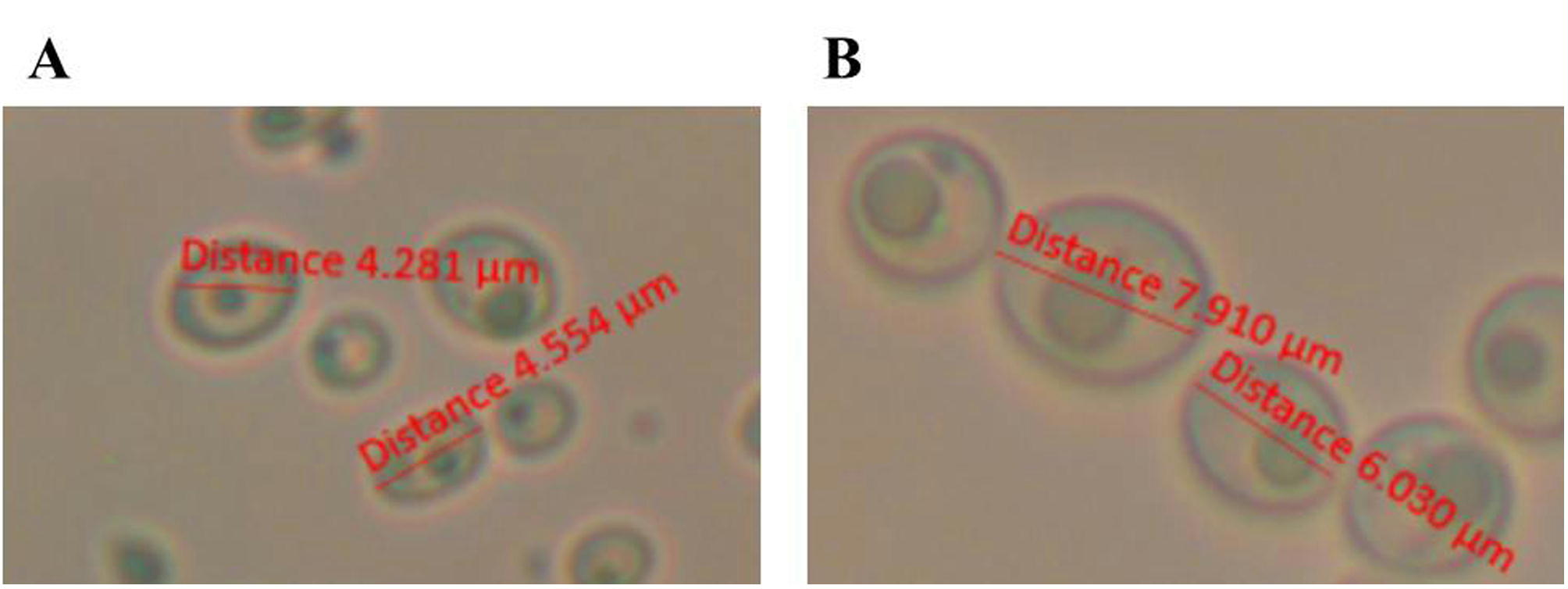

Supplement: Additional file 4: — Figure S3. Flow cytometric analysis of DNA content of Candida species. (JPEG 76 kb) [file 12864_2015_1863_MOESM4_ESM.jpg]
