# Supplementary material for: Draft genome of a commonly misdiagnosed multidrug resistant pathogen Candida auris
Source: BMC Genomics. 2015 Sep 7;16(1):686. doi: 10.1186/s12864-015-1863-z (PMC4562351; doi:10.1186/s12864-015-1863-z)
Supplement: Additional file 5: — Multiple sequence alignment of rRNA and ITS sequences of C. auris 6684 and reported Indian strains of C. auris from NCBI. (PDF 122 kb) [file 12864_2015_1863_MOESM5_ESM.pdf]

```
CLUSTAL 2.1 multiple sequence alignment


VPCI_470/P/14       TTGATATTTTGCATACACACTGATTTGGATTTTTAAACTAACCCAACGTTAAGTTCAACT 60
VPCI_672/P/12       -------------------------------TTTAAACTAACCCAACGTTAAGTTCAACT 29
VPCI_673/P/12       -------------------------------------CTAACCCAACGTTAAGTTCAACT 23
VPCI_676/P/12       --------------------------------------TAACCCAACGTTAAGTTCAACT 22
VPCI_107/P/14       -------------------------------------CTAACCCAACGTTAAGTTCAACT 23
VPCI_711/P/12       -------------------------------------CTAACCCAACGTTAAGTTCAACT 23
VPCI_709/P/12       -------------------------------------CTAACCCAACGTTAAGTTCAACT 23
VPCI_671/P/12       ----------------------------------AAACTAACCCAACGTTAAGTTCAACT 26
VPCI_677/P/12       -------------------------------TTAAAACTAACCCAACGTTAAGTTCAACT 29
6684                TTGATATTTTGCATACACACTGATTTGGATTTTAAAACTAACCCAACGTTAAGTTCAACT 60
VPCI_692/P/12       TTGATATTTTGCATACACACTGATTTGGATTTTAAAACTAACCCAACGTTAAGTTCAACT 60
VPCI_105/P/14       TTGATATTTTGCATACACACTGATTTGGATTTTAAAACTAACCCAACGTTAAGTTCAACT 60
VPCI_106/P/14       TTGATATTTTGCATACACACTGATTTGGATTTTAAAACTAACCCAACGTTAAGTTCAACT 60
VPCI_717/P/14       TTGATATTTTGCATACACACTGATTTGGATTTTAAAACTAACCCAACGTTAAGTTCAACT 60
VPCI_683/P/12       TTGATATTTTGCATACACACTGATTTGGATTTTAAAACTAACCCAACGTTAAGTTCAACT 60
VPCI_1130/P/13      TTGATATTTTGCATACACACTGATTTGGATTTTAAAACTAACCCAACGTTAAGTTCAACT 60
VPCI_670/P/12       TTGATATTTTGCATACACACTGATTTGGATTTTAAAACTAACCCAACGTTAAGTTCAACT 60
VPCI_708/P/12       TTGATATTTTGCATACACACTGATTTGGATTTTAAAACTAACCCAACGTTAAGTTCAACT 60
VPCI_669/P/12       TTGATATTTTGCATACACACTGATTTGGATTTTAAAACTAACCCAACGTTAAGTTCAACT 60
VPCI_712/P/12       TTGATATTTTGCATACACACTGATTTGGATTTTAAAACTAACCCAACGTTAAGTTCAACT 60
                                                          **********************

VPCI_470/P/14       AAAACAAAAACATAAAACTTTCAACAACGGATCTCTTGGTTCTCGC-ATCGATGAAGAAC 119
VPCI_672/P/12       AAAACAAAAACATAAAACTTTCAACAACGGATCTCTTGGTTCTCGC-ATCGATGAAGAAC 88
VPCI_673/P/12       AAAACAAAAACATAAAACTTTCAACAACGGATCTCTTGGTTCTCGC-ATCGATGAAGAAC 82
VPCI_676/P/12       AAAACAAAAACATAAAACTTTCAACAACGGATCTCTTGGTTCTCGC-ATCGATGAAGAAC 81
VPCI_107/P/14       AAAACAAAAACATAAAACTTTCAACAACGGATCTCTTGGTTCTCGCTATCGATGAAGAAC 83
VPCI_711/P/12       AAAACAAAAACATAAAACTTTCAACAACGGATCTCTTGGTTCTCGC-ATCGATGAAGAAC 82
VPCI_709/P/12       AAAACAAAAACATAAAACTTTCAACAACGGATCTCTTGGTTCTCGC-ATCGATGAAGAAC 82
VPCI_671/P/12       AAAACAAAAACATAAAACTTTCAACAACGGATCTCTTGGTTCTCGC-ATCGATGAAGAAC 85
VPCI_677/P/12       AAAACAAAAACATAAAACTTTCAACAACGGATCTCTTGGTTCTCGC-ATCGATGAAGAAC 88
6684                AAAACAAAAACATAAAACTTTCAACAACGGATCTCTTGGTTCTCGC-ATCGATGAAGAAC 119
VPCI_692/P/12       AAAACAAAAACATAAAACTTTCAACAACGGATCTCTTGGTTCTCGC-ATCGATGAAGAAC 119
VPCI_105/P/14       AAAACAAAAACATAAAACTTTCAACAACGGATCTCTTGGTTCTCGC-ATCGATGAAGAAC 119
VPCI_106/P/14       AAAACAAAAACATAAAACTTTCAACAACGGATCTCTTGGTTCTCGC-ATCGATGAAGAAC 119
VPCI_717/P/14       AAAACAAAAACATAAAACTTTCAACAACGGATCTCTTGGTTCTCGC-ATCGATGAAGAAC 119
VPCI_683/P/12       AAAACAAAAACATAAAACTTTCAACAACGGATCTCTTGGTTCTCGC-ATCGATGAAGAAC 119
VPCI_1130/P/13      AAAACAAAAACATAAAACTTTCAACAACGGATCTCTTGGTTCTCGC-ATCGATGAAGAAC 119
VPCI_670/P/12       AAAACAAAAACATAAAACTTTCAACAACGGATCTCTTGGTTCTCGC-ATCGATGAAGAAC 119
VPCI_708/P/12       AAAACAAAAACATAAAACTTTCAACAACGGATCTCTTGGTTCTCGC-ATCGATGAAGAAC 119
VPCI_669/P/12       AAAACAAAAACATAAAACTTTCAACAACGGATCTCTTGGTTCTCGC-ATCGATGAAGAAC 119
VPCI_712/P/12       AAAACAAAAACATAAAACTTTCAACAACGGATCTCTTGGTTCTCGC-ATCGATGAAGAAC 119
                    ********************************************** *************

VPCI_470/P/14       GCAGCGAAATGCGATACGTAGTATGACTTGCAGACGTGAATCATCGAATCTTTGAACGCA 179
VPCI_672/P/12       GCAGCGAAATGCGATACGTAGTATGACTTGCAGACGTGAATCATCGAATCTTTGAACGCA 148
VPCI_673/P/12       GCAGCGAAATGCGATACGTAGTATGACTTGCAGACGTGAATCATCGAATCTTTGAACGCA 142
VPCI_676/P/12       GCAGCGAAATGCGATACGTAGTATGACTTGCAGACGTGAATCATCGAATCTTTGAACGCA 141
VPCI_107/P/14       GCAGCGAAATGCGATACGTAGTATGACTTGCAGACGTGAATCATCGAATCTTTGAACGCA 143
VPCI_711/P/12       GCAGCGAAATGCGATACGTAGTATGACTTGCAGACGTGAATCATCGAATCTTTGAACGCA 142
VPCI_709/P/12       GCAGCGAAATGCGATACGTAGTATGACTTGCAGACGTGAATCATCGAATCTTTGAACGCA 142
VPCI_671/P/12       GCAGCGAAATGCGATACGTAGTATGACTTGCAGACGTGAATCATCGAATCTTTGAACGCA 145
VPCI_677/P/12       GCAGCGAAATGCGATACGTAGTATGACTTGCAGACGTGAATCATCGAATCTTTGAACGCA 148
6684                GCAGCGAAATGCGATACGTAGTATGACTTGCAGACGTGAATCATCGAATCTTTGAACGCA 179
VPCI_692/P/12       GCAGCGAAATGCGATACGTAGTATGACTTGCAGACGTGAATCATCGAATCTTTGAACGCA 179
VPCI_105/P/14       GCAGCGAAATGCGATACGTAGTATGACTTGCAGACGTGAATCATCGAATCTTTGAACGCA 179
VPCI_106/P/14       GCAGCGAAATGCGATACGTAGTATGACTTGCAGACGTGAATCATCGAATCTTTGAACGCA 179
VPCI_717/P/14       GCAGCGAAATGCGATACGTAGAATGACTTGCAGACGTGAATCATCGAATCTTTGAACGCA 179
VPCI_683/P/12       GCAGCGAAATGCGATACGTAGTATGACTTGCAGACGTGAATCATCGAATCTTTGAACGCA 179
VPCI_1130/P/13      GCAGCGAAATGCGATACGTAGTATGACTTGCAGACGTGAATCATCGAATCTTTGAACGCA 179
```

```
VPCI_670/P/12       GCAGCGAAATGCGATACGTAGTATGACTTGCAGACGTGAATCATCGAATCTTTGAACGCA 179
VPCI_708/P/12       GCAGCGAAATGCGATACGTAGTATGACTTGCAGACGTGAATCATCGAATCTTTGAACGCA 179
VPCI_669/P/12       GCAGCGAAATGCGATACGTAGTATGACTTGCAGACGTGAATCATCGAATCTTTGAACGCA 179
VPCI_712/P/12       GCAGCGAAATGCGATACGTAGTATGACTTGCAGACGTGAATCATCGAATCTTTGAACGCA 179
                    ******************** ***************************************

VPCI_470/P/14       CATTGCGCCTTGGGGTATTCCCCAAGGCATGCCTGTTTGAGC-GTGATGTCTTCTCACCA 238
VPCI_672/P/12       CATTGCGCCTTGGGGTATTCCCCAAGGCATGCCTGTTTGAGC-GTGATGTCTTCTCACCA 207
VPCI_673/P/12       CATTGCGCCTTGGGGTATTCCCCAAGGCATGCCTGTTTGAGC-GTGATGTCTTCTCACCA 201
VPCI_676/P/12       CATTGCGCCTTGGGGTATTCCCCAAGGCATGCCTGTTTGAGC-GTGATGTCTTCTCACCA 200
VPCI_107/P/14       CATTGCGCCTTGGGGTATTCCCCAAGGCATGCCTGTTTGAGC-GTGATGTCTTCTCACCA 202
VPCI_711/P/12       CATTGCGCCTTGGGGTATTCCCCAAGGCATGCCTGTTTGAGC-GTGATGTCTTCTCACCA 201
VPCI_709/P/12       CATTGCGCCTTGGGGTATTCCCCAAGGCATGCCTGTTTGAGC-GTGATGTCTTCTCACCA 201
VPCI_671/P/12       CATTGCGCCTTGGGGTATTCCCCAAGGCATGCCTGTTTGAGC-GTGATGTCTTCTCACCA 204
VPCI_677/P/12       CATTGCGCCTTGGGGTATTCCCCAAGGCATGCCTGTTTGAGC-GTGATGTCTTCTCACCA 207
6684                CATTGCGCCTTGGGGTATTCCCCAAGGCATGCCTGTTTGAGC-GTGATGTCTTCTCACCA 238
VPCI_692/P/12       CATTGCGCCTTGGGGTATTCCCCAAGGCATGCCTGTTTGAGC-GTGATGTCTTCTCACCA 238
VPCI_105/P/14       CATTGCGCCTTGGGGTATTCCCCAAGGCATGCCTGTTTGAGC-GTGATGTCTTCTCACCA 238
VPCI_106/P/14       CATTGCGCCTTGGGGTATTCCCCAAGGCATGCCTGTTTGAGC-GTGATGTCTTCTCACCA 238
VPCI_717/P/14       CATTGCGCCTTGGGGTATTCCCCAAGGCATGCCTGTTTGAGC-GTGATGTCTTCTCACCA 238
VPCI_683/P/12       CATTGCGCCTTGGGGTATTCCCCAAGGCATGCCTGTTTGAGC-GTGATGTCTTCTCACCA 238
VPCI_1130/P/13      CATTGCGCCTTGGGGTATTCCCCAAGGCATGCCTGTTTGAGC-GTGATGTCTTCTCACCA 238
VPCI_670/P/12       CATTGCGCCTTGGGGTATTCCCCAAGGCATGCCTGTTTGAGC-GTGATGTCTTCTCACCA 238
VPCI_708/P/12       CATTGCGCCTTGGGGTATTCCCCAAGGCATGCCTGTTTGAGCCGTGATGTCTTCTCACCA 239
VPCI_669/P/12       CATTGCGCCTTGGGGTATTCCCCAAGGCATGCCTGTTTGAGC-GTGATGTCTTCTCACCA 238
VPCI_712/P/12       CATTGCGCCTTGGGGTATTCCCCAAGGCATGCCTGTTTGAGC-GTGATGTCTTCTCACCA 238
                    ****************************************** *****************

VPCI_470/P/14       ATCTTCGCGGTGGCGTTGCATTCACAAAATTACAGCTTGCACGAAAAAAATCTACGCTTT 298
VPCI_672/P/12       ATCTTCGCGGTGGCGTTGCATTCACAAAATTACAGCTTGCACGAAAAAAATCTACGCTTT 267
VPCI_673/P/12       ATCTTCGCGGTGGCGTTGCATTCACAAAATTACAGCTTGCACGAAAAAAATCTACGCTTT 261
VPCI_676/P/12       ATCTTCGCGGTGGCGTTGCATTCACAAAATTACAGCTTGCACGAAAAAAATCTACGCTTT 260
VPCI_107/P/14       ATCTTCGCGGTGGCGTTGCATTCACAAAATTACAGCTTGCACGAAAAAAATCTACGCTTT 262
VPCI_711/P/12       ATCTTCGCGGTGGCGTTGCATTCACAAAATTACAGCTTGCACGAAAAAAATCTACGCTTT 261
VPCI_709/P/12       ATCTTCGCGGTGGCGTTGCATTCACAAAATTACAGCTTGCACGAAAAAAATCTACGCTTT 261
VPCI_671/P/12       ATCTTCGCGGTGGCGTTGCATTCACAAAATTACAGCTTGCACGAAAAAAATCTACGCTTT 264
VPCI_677/P/12       ATCTTCGCGGTGGCGTTGCATTCACAAAATTACAGCTTGCACGAAAAAAATCTACGCTTT 267
6684                ATCTTCGCGGTGGCGTTGCATTCACAAAATTACAGCTTGCACGAAAAAAATCTACGCTTT 298
VPCI_692/P/12       ATCTTCGCGGTGGCGTTGCATTCACAAAATTACAGCTTGCACGAAAAAAATCTACGCTTT 298
VPCI_105/P/14       ATCTTCGCGGTGGCGTTGCATTCACGAAATTACAGCTTGCACGAAAAAAATCTACGCTTT 298
VPCI_106/P/14       ATCTTCGCGGTGGCGTTACATTCACAAAATTACAGCTTGCACGAAAAAAATCTACGCTTT 298
VPCI_717/P/14       ATCTTCGCGGTGGCGTTGCATTCACAAAATTACAGCTTGCACGAAAAAAATCTACGCTTT 298
VPCI_683/P/12       ATCTTCGCGGTGGCGTTGCATTCACAAAATTACAGCTTGCACGAAAAAAATCTACGCTTT 298
VPCI_1130/P/13      ATCTTCGCGGTGGCGTTGCATTCACAAAATTACAGCTTGCACGAAAAAAATCTACGCTTT 298
VPCI_670/P/12       ATCTTCGCGGTGGCGTTGCATTCACAAAATTACAGCTTGCACGAAAAAAATCTACGC--- 295
VPCI_708/P/12       ATCTTCGCGGTGGCGTTGCATTCACAAAATTACAGCTTGCACGAAAAAAATCTACGCTT- 298
VPCI_669/P/12       ATCTTCGCGGTGGCGTTGCATTCACAAAATTACAGCTTGCACGAAAAAAATCTACGC--- 295
VPCI_712/P/12       ATCTTCGCGGTGGCGTTGCATTCACAAAATTACAGCTTGCACGAAAAAAATCTACGCTT- 297
                    ***************** ******* *******************************

VPCI_470/P/14       TTTTTTCGTTTTGTTGTCGCC-TCAAATCAGGTAGGACTACCCGCTGAACTTAA 351
VPCI_672/P/12       TTTTTTCGTTTTGTTGTCGCC-TCAAATCAGGTAGGACTACCCGCTGAACTTAA 320
VPCI_673/P/12       TTTTTTCGTTTTGTTGTCGCC-TCAAATCAGGTAGGACTACCCGCTGAACTTAA 314
VPCI_676/P/12       TTTTTTCGTTTTGTTGTCGCC-TCAAATCAGGTAGGACTACCCGCTGAACTTAA 313
VPCI_107/P/14       TTTTTTCGTTTTGTTGTCGCC-TCAAATCAGGTAGGACTACCCGCTGAACTTAA 315
VPCI_711/P/12       TTTTTTCGTTTTGTTGTCGCC-TCAAATCAGGTAGGACTACCCGCTGAACTTAA 314
VPCI_709/P/12       TTTTTTCGTTTTGTTGTCGCC-TCAAATCAGGTAGGACTACCCGCTGAACTTAA 314
VPCI_671/P/12       TTTTTTCGTTTTGTTGTCGCC-TCAAATCAGGTAGGACTACCCGCTGAACTTAA 317
VPCI_677/P/12       TTTTTTCGTTTTGTTGTCGCC-TCAAATCAGGTAGGACTACCCGCTGAACTTAA 320
6684                TTTTTTCGTTTTGTTGTCGCC-TCAAATCAGGTAGGACTACCCGCTGAACTTAA 351
VPCI_692/P/12       TTTTTTCGTTTTGTTGTCGCCCTCAAATCAGGTAGGACTACCCGCTGAACTTAA 352
VPCI_105/P/14       TTTTTTCGTTTTGTTGTCGCC-TCAAATCAGGTAGGACTACCCGCTGAACTTAA 351
VPCI_106/P/14       TTTTTTCGTTTTGTTGTCGCC-TCAAATCAGGTAGGACTACCCGCTGAACTTAA 351
```

```
VPCI_717/P/14       TTTTTTCGTTTTGTTGTCGCC-TCAAATCAGGTAGGACTACCCGCTGAACTTAA 351
VPCI_683/P/12       TTTTTTCGTTTTGTTGTCGCC-TCAAATCAGGTAGGACTACCCGCTGAACTTAA 351
VPCI_1130/P/13      TTTTTTCGTTTTGTTGTCGCC-TCAAATCAGGTAGGACTACCCGCTGAACTTAA 351
VPCI_670/P/12       ------------------------------------------------------
VPCI_708/P/12       ------------------------------------------------------
VPCI_669/P/12       ------------------------------------------------------
VPCI_712/P/12       ------------------------------------------------------
```
